# Generation of a Soluble Form of Human Endoglin Fused to Green Fluorescent Protein

**DOI:** 10.3390/ijms222011282

**Published:** 2021-10-19

**Authors:** Lidia Ruiz-Llorente, M. Cristina Vega, Francisco J. Fernández, Carmen Langa, Nicholas W. Morrell, Paul D. Upton, Carmelo Bernabeu

**Affiliations:** 1 Centro de Investigaciones Biológicas Margarita Salas, Consejo Superior de Investigaciones Científicas (CSIC), 28040 Madrid, Spain; lidia.ruizl@uah.es (L.R.-L.); cvega@cib.csic.es (M.C.V.); fjfernandez@abvance.com (F.J.F.); mcarmenlangap@gmail.com (C.L.); 2 Centro de Investigación Biomédica en Red de Enfermedades Raras (CIBERER), 28040 Madrid, Spain; 3 Biochemistry and Molecular Biology Unit, Department of System Biology, School of Medicine and Health Sciences, University of Alcalá, Alcalá de Henares, 28871 Madrid, Spain; 4 Department of Medicine, University of Cambridge, Addenbrooke’s Hospital, Cambridge CB2 0QQ, UK; nwm23@cam.ac.uk (N.W.M.); pdu21@medschl.cam.ac.uk (P.D.U.)

**Keywords:** endoglin, GFP, soluble endoglin, fusion protein, recombinant protein, fluorescence, BMP, TGF-β, endothelium

## Abstract

Endoglin (Eng, CD105) is a type I membrane glycoprotein that functions in endothelial cells as an auxiliary receptor for transforming growth factor β (TGF-β)/bone morphogenetic protein (BMP) family members and as an integrin ligand, modulating the vascular pathophysiology. Besides the membrane-bound endoglin, there is a soluble form of endoglin (sEng) that can be generated by the action of the matrix metalloproteinase (MMP)-14 or -12 on the juxtamembrane region of its ectodomain. High levels of sEng have been reported in patients with preeclampsia, hypercholesterolemia, atherosclerosis and cancer. In addition, sEng is a marker of cardiovascular damage in patients with hypertension and diabetes, plays a pathogenic role in preeclampsia, and inhibits angiogenesis and tumor proliferation, migration, and invasion in cancer. However, the mechanisms of action of sEng have not yet been elucidated, and new tools and experimental approaches are necessary to advance in this field. To this end, we aimed to obtain a fluorescent form of sEng as a new tool for biological imaging. Thus, we cloned the extracellular domain of endoglin in the pEGFP-N1 plasmid to generate a fusion protein with green fluorescent protein (GFP), giving rise to pEGFP-N1/Eng.EC. The recombinant fusion protein was characterized by transient and stable transfections in CHO-K1 cells using fluorescence microscopy, SDS-PAGE, immunodetection, and ELISA techniques. Upon transfection with pEGFP-N1/Eng.EC, fluorescence was readily detected in cells, indicating that the GFP contained in the recombinant protein was properly folded into the cytosol. Furthermore, as evidenced by Western blot analysis, the secreted fusion protein yielded the expected molecular mass and displayed a specific fluorescent signal. The fusion protein was also able to bind to BMP9 and BMP10 in vitro. Therefore, the construct described here could be used as a tool for functional in vitro studies of the extracellular domain of endoglin.

## 1. Introduction

Human endoglin is a type I integral membrane protein with a large (561 amino acids) glycosylated extracellular region, a single hydrophobic transmembrane domain, and a short cytosolic domain [1]. It is predominantly expressed on proliferating endothelial cells and plays a critical role in vascular pathophysiology [2,3,4,5,6]. In addition to the membrane-bound form, a soluble form of endoglin (sEng) can be generated by the action of the metalloproteases (MMP) MMP-14 or MMP-12 on the ectodomain of the membrane protein [7,8,9,10]. Abnormal levels of sEng were found in several endothelium-related pathological conditions, including preeclampsia [11,12], hypercholesterolemia and atherosclerosis [5,13], diabetes mellitus [14], hypertension [15], diabetic retinopathy [16], coronary artery disease [17], hereditary hemorrhagic telangiectasia [18,19], acute myocardial infarction [20] and cancer [21,22]. In some of these diseases, sEng has been postulated to be a biomarker for severity correlation and prognosis.

Many of the cellular functions of endoglin are ascribed to the extracellular region, including its capacity to bind members of the transforming growth factor-β (TGF-β), TGF-β signaling receptors types I and II, or integrin family members [23,24,25,26,27]. Because the extracellular region encompasses ~90% of the human endoglin protein [1] and most of the cellular functions of endoglin map to the extracellular domain [2,5], some studies focused on the recombinant expression of this region as a soluble protein. Early studies achieved the bacterial expression of different fragments of the endoglin ectodomain, but the resulting recombinant proteins were in inclusion bodies [28]. Because human endoglin undergoes post-translational modifications, including glycosylation and phosphorylation [1,29], the lack of these modifications in a bacterial expression system might lead to protein aggregation and targeting to inclusion bodies. By contrast, several studies reported the mammalian expression in vitro and in vivo of different soluble active forms of the extracellular region of endoglin, which served to investigate its function in angiogenesis, wound healing, endothelial dysfunction, hereditary hemorrhagic telangiectasia, and hypertension, among others [5,19,25,26,30,31].

Endoglin is expressed as a disulfide-linked homodimer [1,32]. Structurally, the extracellular region of endoglin contains a juxtamembrane zona pellucida (ZP) domain of 260 amino acid residues and an orphan domain, which does not show any significant homology to any other protein family and is located in the NH2-terminus of the protein [33]. The ZP domain encodes an Arg–Gly–Asp (RGD) tripeptide that partly mediates the endoglin interaction with integrins [34,35,36,37]. In contrast, the orphan domain accounts for the binding to bone morphogenetic protein 9 (BMP9) and BMP10, in agreement with the role of endoglin as an auxiliary TGF-β receptor [24,25,26,38,39]. In addition, several studies identified novel endoglin-specific interactors, suggesting the involvement of the extracellular region of endoglin in TGF-β- and integrin-independent pathways [40,41]. Several intracellular ligands of the endoglin ectodomain, including tripartite motif-containing protein 21 (TRIM21), an E3 ubiquitin-protein ligase, and galectin-3, a secreted member of the lectin family, were identified [41].

Although recent studies have provided new insights for understanding the wide range of pathophysiological effects of sEng, the underlying mechanisms of action of the extracellular region of endoglin are yet to be clarified [19,42,43]. This is partly due to the lack of new and appropriate reagents and experimental approaches. Previous reports showed that expression of the whole extracellular domain of endoglin (Glu26–Gly586) results in a soluble and functional protein with the capacity to bind BMP9 and BMP10 [25,26]. Here, we generated a soluble protein containing the same endoglin ectodomain fused with the enhanced green fluorescent protein (EGFP), a derivative of GFP. Fluorescent proteins are versatile and indispensable biological markers for tracking protein localization or monitoring cellular and physiological processes [44,45,46]. Specifically, this technology was widely used to study other secreted proteins [47,48,49]. Among the advantages of using a fluorescent sEng is the capacity to track its binding to specific cell surface receptors or the identification of novel interactors within the extracellular or intracellular compartment, including those along the secretory pathway. For example, these studies can be achieved by direct fluorescent visualization using cellular microscopy or Förster resonance energy transfer (FRET) for protein–protein interaction analysis. With these studies of the sEng interactome, we intend to improve our understanding of sEng function. Here, we generated an expression vector encoding the extracellular domain of endoglin fused with EGFP (pEGFP–N1/ENG.EC). The recombinant protein was characterized by transient and stable transfections in mammalian cells using fluorescence microscopy, flow cytometry, Western blot, and ELISA techniques. The capacity to bind BMP9 and BMP10 was also confirmed in vitro. Results suggest that this newly described reagent may be a valuable tool to study the role of the sEng network in different pathophysiological conditions.

## 2. Results

**Figure 1:**
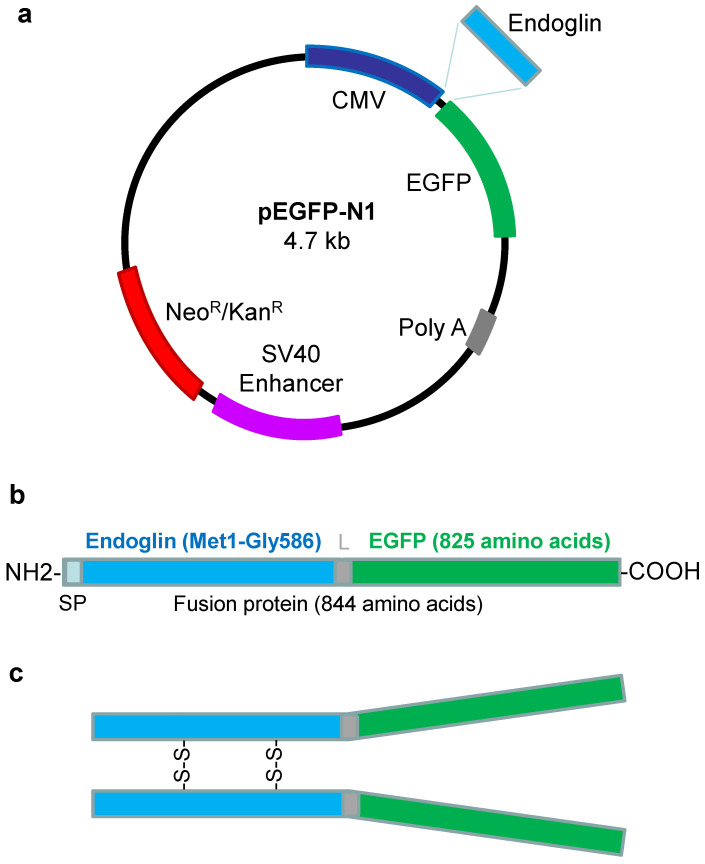
Generation of expression vector encoding endoglin–GFP fusion protein. (**a**) Schematic representation of pEGFP–N1 expression vector containing a cytomegalovirus promoter, the neomycin/kanamycin resistance gene, an SV40 enhancer, and a polyA sequence. Human endoglin cDNA (Met1-Gly586) was inserted into the multicloning site upstream of EGFP cDNA. (**b**) Schematic representation of predicted primary structure of the fusion protein. Relative positions of endoglin (amino terminus), a small linker (L), and EGFP (carboxy terminus) sequences within the fusion protein that contains a total of 844 amino acids are indicated. SP, Signal peptide. (**c**) Schematic representation of predicted fusion protein upon secretion. Presence of endoglin disulfide linkages (S-S) leading to a dimeric protein is indicated. Potential glycosylation sites were omitted for simplification. Drawings are not to scale.

In order to obtain a fusion protein between endoglin and the enhanced green fluorescent protein (EGFP), we cloned the extracellular domain of endoglin in expression vector pEGFP–N1 to generate the pEGFP–N1/Eng.EC plasmid (Figure 1a). The resulting construct encodes the ectodomain of endoglin (Glu26–Gly586), preceded by its native export signal peptide at the N-terminus, followed in-frame by EGFP at the C-terminus, leading to a fusion protein of 844 amino acids (Figure 1b). Once secreted, the predicted fusion protein is expected to be a disulfide-linked dimer (Figure 1c) in agreement with the dimeric nature of endoglin [1].

**Figure 2:**
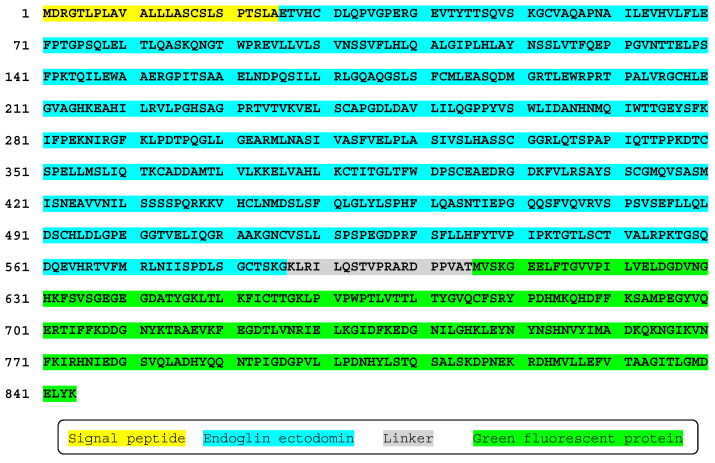
Predicted amino acid sequence of endoglin–GFP fusion protein. Amino acids are numbered on the left. Positions of endoglin signal peptide at N terminus, endoglin extracellular region, a linker sequence, and the green fluorescent protein (GFP) at the C terminus are indicated. A single-letter code for amino acids is used.

The predicted sequence of the fusion protein shows the endoglin signal peptide of 25 amino acids, followed by 561 residues of the whole extracellular region of endoglin, a linker fragment of 19 amino acids, and 239 residues of GFP (Figure 2).

**Figure 3:**
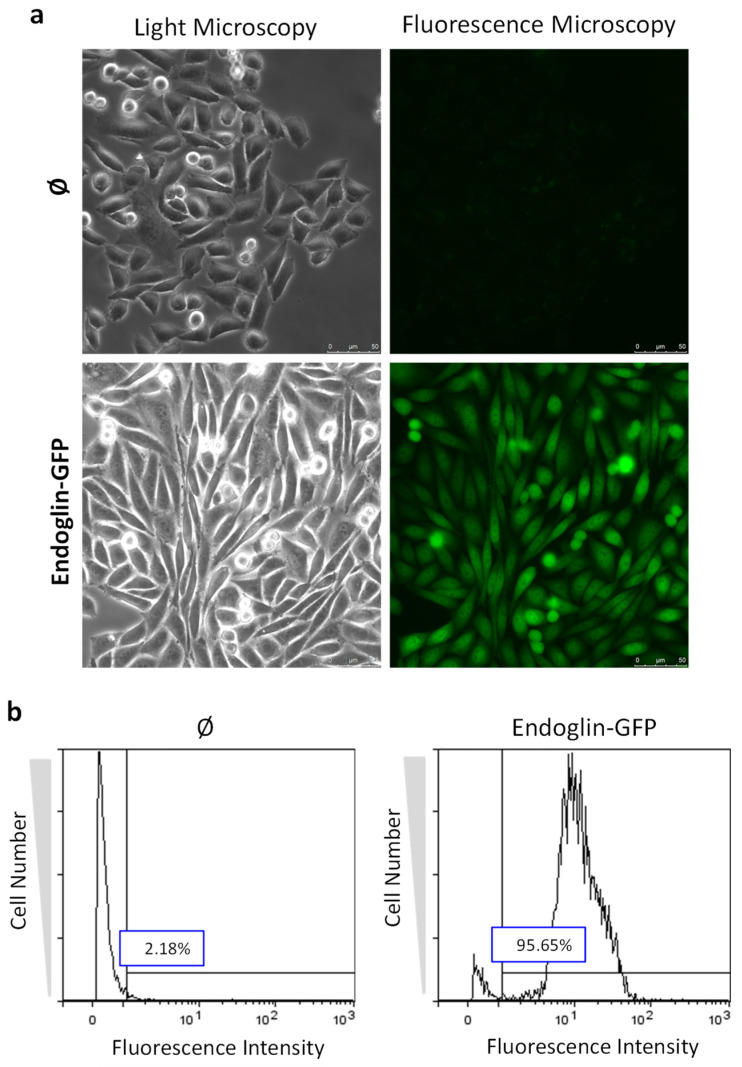
Fluorescence characterization of cell transfectants expressing endoglin–GFP fusion protein. CHO-K1 cells stably transfected with pEGFP–N1/Eng.EC (Endoglin-GFP) or an empty vector (Ø) were analyzed by (**a**) fluorescence microscopy or (**b**) flow cytometry. (**a**) Cells were seeded and cultured on 35 mm glass bottom dishes. After removing media and washing with PBS, cells were observed under fluorescence and light microscopy using a multidimensional microscope (System Leica AF6000 LX, Leica Microsystems, Wetzlar, Germany) at 40× magnification. (**b**) Cells were trypsinized, washed, and resuspended in PBS. Cell fluorescence was analyzed with a Coulter EPICS-XL flow cytometer using logarithmic amplifiers. Vertical line represents the gate determined by the intensity corresponding to the empty vector (Ø). Percentage of positive cells indicated by the horizontal bar.

Next, we transfected the pEGFP–N1/Eng.EC vector in CHO-K1 cells, and, after stringent selection in the presence of the G418 antibiotic, a stable cell line was obtained. Parallel transfection experiments using an empty vector were also carried out to generate a stable cell line used as a negative control. To validate the expression of the recombinant endoglin–GFP fusion protein, fluorescence characterization of cell transfectants was carried out (Figure 3). As shown in Figure 3a, a clear green fluorescence signal was observed in CHO-K1 cells transfected with the pEGFP–N1/Eng.EC vector, as evidenced by fluorescence microscopy. By contrast, control cells transfected with the empty vector did not yield any significant fluorescence. In addition, fluorescence was readily detected in CHO-K1/pEGFP-N1/Eng.EC cells by flow cytometry analysis, showing that more than 95% of the cells were positive for EGFP, compared to 2% of the negative control (Figure 3b).

**Figure 4:**
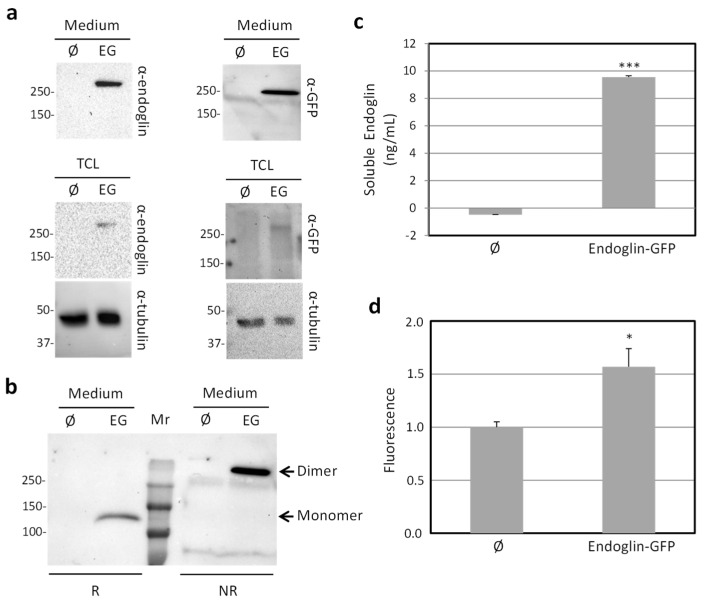
Expression of endoglin–GFP fusion protein by cell transfectants. CHO-K1 cells stably transfected with pEGFP–N1/Eng.EC (EG) or an empty vector (Ø) were cultured for 3 days. (**a**,**b**) Western blot analysis. Proteins contained in the culture medium or total cell lysates (TCL) were separated by SDS-PAGE under reducing (R) or nonreducing (NR) conditions and transferred to PDVF membranes. Immunodetection by chemiluminescence of endoglin or GFP was carried out with specific primary antibodies, using α-tubulin as a loading control. Positions of molecular mass markers are indicated (in kDa) on the left. (**a**) Samples were run under NR conditions. (**b**) Immunodetection was carried out with anti-GFP antibodies, and positions of the dimer and monomer are indicated. Mr, molecular weight reference. (**c**,**d**) Culture medium from CHO-K1 cell transfectants analyzed by ELISA to (**c**) measure concentrations of soluble endoglin in a GloMax multidetection system and (**d**) determine fluorescence in a Varioskan instrument at 488/509 nm. *p* < 0.01 (*) and *p* < 0.005 (***).

The characterization of the recombinant protein was performed by Western blot analysis using antibodies to either endoglin or GFP (Figure 4a). Culture supernatants from CHO-K1 cells transfected with the pEGFP–N1/Eng.EC vector revealed the presence of a soluble recombinant protein with a molecular weight of ~275 kDa. A signal with the same molecular mass was also observed in total cell extracts, but in this case, the signal was weaker than that in the supernatants (Figure 4a). This is likely due to the scarce and transient presence of the fusion protein along the secretory pathway and the nuclear translocation of EGFP (Figure 3a), as reported in mammalian cells [50]. Western blot analysis of the secreted protein, previously subjected to reducing or nonreducing conditions, demonstrated that it is expressed as a disulfide-linked homodimer (Figure 4b). This agrees with the dimeric nature of endoglin due to cysteine residues in its extracellular region involved in interchain disulfide bonding [1,30,32,39]. The presence of the fusion protein in the culture medium was confirmed by using specific ELISA for endoglin (Figure 4c) and by measuring its fluorescence (Figure 4d). The average protein yield obtained in the different culture supernatants was within the concentration range of 5.0–20 ng/mL. These results suggest that the secreted fusion protein was correctly expressed in a fluorescently active form.

**Figure 5:**
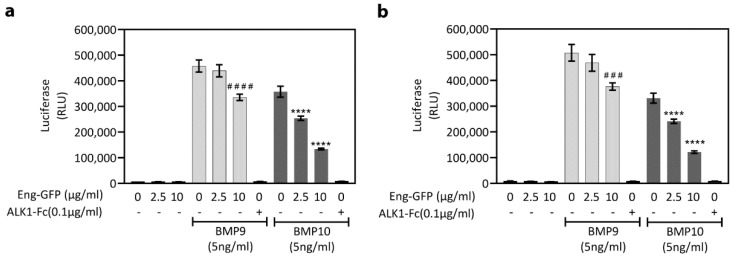
Endoglin–GFP (Eng–GFP) fusion protein attenuates BMP9 and BMP10 signaling in C2C12-BRE cells. BMP9 or BMP10 (each 5 ng/mL) were preincubated with endoglin–GFP (2.5 µg/mL or 10 µg/mL) or ALK1-Fc (100 ng/mL) for 18 h at either (**a**) RT or (**b**) 37 °C. Mixes were then added to C2C12-BRE cells (*n* = 4 wells/treatment) for 6 h, followed by cell lysis with One-GLO™ reagent and luciferase activity measurement. Data are representative of *n* = 3 separate experiments. ^###^ *p* < 0.0005 and ^####^ *p* < 0.0001 relative to BMP9, and **** *p* < 0.0001 relative to BMP10, one-way ANOVA.

The biological activity of the endoglin–GFP fusion protein was assessed by examining its capacity to neutralize BMP9 and BMP10 signaling in the C2C12-BRE reporter line [51]. Preincubation of the purified endoglin–GFP protein (10 µg/mL) with BMP9 or BMP10 significantly attenuated the luciferase response in the C2C12-BRE cells (Figure 5). At 2.5 µg/mL, endoglin–GFP significantly inhibited the response to BMP10, whereas the BMP9 response was not significantly reduced. These results confirmed that the endoglin–GFP construct could bind to BMP9 and BMP10.

## 3. Discussion

Soluble endoglin encompasses most of the ectodomain of membrane-bound endoglin, and is involved in several cardiovascular and metabolic pathologies [2,5,52]. While the cellular expression and functional role of membrane endoglin were widely studied and characterized [3,5,53,54,55], the cellular and molecular mechanisms by which sEng exerts its pathophysiological effects are poorly understood [19,42,43]. Thus, the generation of new sEng-related reagents may be useful in evolving technologies to advance the scientific knowledge of this soluble protein. Whereas a previous study reported the expression of a full-length membrane-bound endoglin fused to a fluorescent protein [56], to our knowledge, we generated for the first time a fusion protein here between the whole extracellular region of endoglin and GFP. This recombinant fusion protein is expressed as a disulfide-linked dimer of 275 kDa and is readily secreted by CHO-K1 cell transfectants, as evidenced by Western blot and ELISA techniques. The GFP fluorescence activity of this fusion protein could be detected in both intracellular and soluble forms. Previous studies suggested that the GFP properly folded, and emitting fluorescence cannot be secreted out of cells without a signal peptide [57,58]. In fact, most pre-existing secretory GFP-derived proteins were generated by a fusion of nascent GFP to the protein of interest that is naturally secreted out of cells [58,59,60]. Thus, the endoglin signal peptide of this construct facilitated the secretion of the functionally active GFP. Nonetheless, the expression of this fluorescent endoglin/GFP fusion protein could be further maximized by screening a mammalian signal-peptide library, as demonstrated with heterologous proteins expressed in bacteria [61]. In addition, the cytotoxicity of GFP from the hydrozoan jellyfish *Aequorea victoria*, when expressed into mammalian cells, should be taken into account to optimize the expression of this GFP fusion protein [62]. Accordingly, long-term cultures of cells expressing GFP fusion proteins may cause cell lysis and proteolysis, resulting both in impaired expression and the leakage of a proteolytically degraded recombinant protein into the medium. This limitation was not observed in our cellular expression system, as we found neither increased cell lysis nor a proteolytically degraded recombinant protein.

The construct described here could be used as a tool for the functional in vitro studies of the extracellular domain of endoglin aiming to study the cell surface receptors of sEng, and identify novel sEng interactors of the extracellular and intracellular proteome [2,25,26,39,40,41]. GFP is highly stable, facilitating the accumulation of GFP fusion proteins and their easy detection in cells. Overall, GFP-derived proteins offer several advantages for in vitro studies. They allow for the direct visual detection of proteins in living cells by fluorescence microscopy, with relatively low background, high resolution, and avoidance of fixation artifacts. The fluorescence activity of GFP fusion proteins in solution can also be detected by fluorometry. At variance with other enzymatic markers, such as luciferase or secreted embryonic alkaline phosphatase (SEAP), GFP fusion proteins do not need substrates, cofactors or complex reactions for marker detection, and can be visualized in living cells or organisms [44,45,63].

The endoglin–GFP fusion protein generated here has a molecular weight of 275 kDa. The large size of this protein might be an advantage for structural studies. The 3D crystal structures at 2.4 Å resolution or better of the individual N-terminal orphan region and the C-terminal bipartite zona pellucida module (ZP) of the endoglin ectodomain were elucidated by X-ray crystallography [39]. However, the whole endoglin ectodomain was only analyzed at 25 Å resolution using single-particle electron microscopy [33], while studies to obtain its crystal structure have so far been unsuccessful. Recently, high-resolution single-particle cryoelectron microscopy (cryo-EM) [64,65] has emerged as a powerful tool for investigating protein structures at resolutions previously only attainable by X-ray crystallography, and whose optimal molecular size range theoretically fits well with the molecular weight of the endoglin–GFP fusion protein described here. Thus, it is interesting to analyze by cryo-EM the secreted endoglin–GFP protein construct.

Previous reports demonstrated that the extracellular domain of endoglin (Glu26–Gly586) can bind BMP9 and BMP10 [25,26,43]. To confirm that the endoglin–GFP protein was functionally active, this protein can inhibit BMP9 and BMP10 signaling in C2C12-BRE cells [51].

Further studies are needed to investigate the utility of this fluorescent construct regarding the structure, protein–protein interactions, and function of sEng, which is critically involved in several cardiovascular diseases.

## 4. Materials and Methods

### 4.1. Plasmids

DNA segments encoding the extracellular domain of human endoglin from amino acid Met1 (position 419) to Gly586 (position 2176) were generated by PCR using PfuTurbo DNA polymerase (Stratagene, La Jolla, CA, USA) and cloned into the pEGFP–N1 expression vector (Clontech, Takara Bio Europe SAS, Saint-Germain-en-Laye, France) inframe with the EGFP protein from *Aequorea victoria* (Figure 1). Briefly, PCR was carried out using pcEXV-ENG [32] as a template to generate the extracellular domain and in the presence of sequence-specific oligonucleotides with add-on sequences surrounded by *NheI* and *HindIII* sites (5′-ATA GCT AGC ATG GAC CGC GGC ACG CTC C -3′and 5′-CGC AAG CTT GCC TTT GCT TGT GCA ACC AGA -3′, respectively). The resulting fragments were double-digested with *NheI* and *HindIII*, and inserted at the *NheI*/*HindIII* sites of the pEGFP–N1 expression vector (Figure 1). The construct (pEGFP–N1/Eng.EC) was verified by DNA sequence analysis. For protein purification purposes, a streptavidin tag (Strep-tag^®^II; WSHPQFEK) was added to the C terminus (GenScript Biotech, Leiden, The Netherlands) leading to the EGFP–N1–EndoglinCDS1 plasmid. In addition, plasmid pcDNA3.1 (ThermoFisher Scientific, Waltham, MA, USA), encoding the neomycin resistance gene was used as a negative control in cell transfections.

### 4.2. Cell Culture and Transfection

The Chinese Hamster Ovary (CHO)-derived cell line CHO-K1 was cultured at 37 °C and 5% CO_2_ in a DMEM culture medium containing 10% heat-inactivated fetal bovine serum (FBS), 2 mM glutamine, and 100 U/mL penicillin/streptomycin. Cells were seeded onto 6-well culture plates, and when they had reached 70–80% confluency by the next day, they were transfected with expression vector pEGFP–N1/Eng.EC or the pcDNA3.1 empty vector (Ø) using Lipofectamine 2000 (Thermo Fisher Scientific, Waltham, MA, USA), according to the manufacturer’s instructions. Two days later, cells were selected first using 1 mg/mL G418 for 3 days, and then 0.8 mg/mL G418 for 5 more days. Next, 0.5 mg/mL G418 was used for the maintenance of stably transfected cell lines. Lastly, EGFP-expressing cells were enriched by cell sorting using Cell Sorter FACS Vantage equipment (BD Biosciences, San Jose, CA, USA).

### 4.3. In Vivo Cell Imaging and Flow Cytometry

For in vivo microscopic analysis, CHO-K1 cells were seeded onto 35 mm glass-bottom dishes. After removing the media and adding PBS, cells were observed by fluorescence and light microscopy using a multidimensional microscope (System Leica AF6000 LX, Leica Microsystems, Wetzlar, Germany) at 40× magnification. For flow cytometry, CHO-K1 cells expressing the endoglin–GFP fusion protein were trypsinized, washed twice with PBS, and resuspended in PBS (500,000 cells/mL). Cell fluorescence was estimated with a Coulter EPICS-XL flow cytometer using logarithmic amplifiers, and results were analyzed with Flow Logic software.

### 4.4. Western Blot Analysis

CHO-K1 cells were centrifuged, and their pellets were resuspended in a high-salt buffer (HSB) containing 0.4 M KCl, 1% NP-40, 10% glycerol, 50 mM Hepes, pH 7.5, and protease inhibitors. The mixture was vortexed, disrupted by sonication, and the remaining cell debris was discarded by centrifugation. Proteins from total cell lysates (30–40 μg) or 3-day cell culture media (30 µL) were separated by SDS-PAGE under reducing or nonreducing conditions and transferred to PVDF membranes (Invitrogen, Waltham, MA, USA). Membranes were blocked with 5% BSA and incubated overnight at 4 °C with primary monoclonal antibodies rabbit anti-endoglin (EPR10145; 1:1000 dilution, Abcam, Cambridge, UK) and mouse anti-GFP (#632380; 1:1000 dilution, Clontech, Takara Bio Europe SAS, Saint-Germain-en-Laye, France). Loading control antibodies were also included (mouse mAb anti-α-tubulin; DM1A, Sigma-Aldrich, St. Louis, MO, USA). Then, membranes were incubated with the corresponding secondary antibodies coupled to horseradish peroxidase (HRP), goat anti-rabbit (P0448; 1:1000 dilution, Dako, Santa Clara, CA, USA) or rabbit anti-mouse (P0260; 1:1000 dilution, Dako, Santa Clara, CA, USA) for 1 h at room temperature. Protein bands were revealed using SuperSignal™ West Pico Plus chemiluminescent substrate (#34580, Thermo Fisher Scientific, Waltham, MA, USA), following the manufacturer’s protocol. Bands were visualized using the Molecular Imager^®^ Gel DocTM XR + System (Bio-Rad, Hercules, CA, USA).

### 4.5. Fluorescence and ELISA and of Secreted Proteins

After 3 days in culture, the medium from CHO-K1 cells was used to measure GFP fluorescence and endoglin protein concentration. Fluorescence in cell culture media was measured using a Varioskan instrument (Thermo Fisher Scientific, Waltham, MA, USA) at 488/509 nm. Concentrations of endoglin in culture supernatants were determined by ELISA, according to the manufacturer´s protocol using the Human Endoglin/CD105 Quantikine kit (DNDG00, R&D Systems, Minneapolis, MN, USA). These immunoassays were measured in a GloMax multidetection system (Promega, Madison, WI, USA).

### 4.6. C2C12-BRE Luciferase Assay

C2C12-BRE cells [51] were seeded at 30,000 cells/well in 96-well plates and grown for 3 days in DMEM containing 10% heat-inactivated FBS and antibiotic/antimycotic (Thermo Fisher Scientific, Waltham, MA, USA). Cells were then washed once with DMEM containing 0.1% FBS and antibiotic/antimycotic; after aspiration of the washed, they were incubated in fresh 0.1% FBS overnight. The endoglin–GFP protein was expressed using the EGFP–N1–EndoglinCDS1 plasmid and purified with Strep-Tactin^®^XT (GenScript Biotech, Leiden, The Netherlands). As the provided dilution of the protein stock (30 µg/mL) was limiting, media for the incubations were prepared using 10X M199 (Sigma-Aldrich, St. Louis, MO, USA). Dilutions of BMP9 or BMP10 (both from R&D Systems, Minneapolis, MN, USA) were incubated in the absence or presence of endoglin–GFP in M199 containing 1.5 mg/mL sodium bicarbonate, 4 mM L-glutamine, 25 mM Hepes, and 0.5% recombinant human serum albumin (Sigma-Aldrich, St. Louis, MO, USA). ALK1-Fc (R&D Systems, Minneapolis, MN, USA) was included as a positive control.

### 4.7. Quantification and Statistical Analysis

All assays were performed in triplicate, each repeated at least twice. Values are expressed as mean ± standard error of the mean (SEM). Direct group–group comparisons were carried out using independent Student’s t-tests; *p* < 0.01 (*) and *p* < 0.05 (**) were considered to be statistically significant, and *p* < 0.005 (***) was considered to be highly statistically significant. For the luciferase assays, data for endoglin–GFP protein were compared by one-way ANOVA to the respective ligand; *p* < 0.0005 (^###^) relative to BMP9 and *p* < 0.0001 (****) relative to BMP10 were considered to be highly significant.

## Data Availability

Data presented in this study are available on request from the corresponding author.
